# Prevention of Rotavirus Diarrhea in Suckling Rats by a Specific Fermented Milk Concentrate with Prebiotic Mixture

**DOI:** 10.3390/nu11010189

**Published:** 2019-01-18

**Authors:** Maria del Mar Rigo-Adrover, Karen Knipping, Johan Garssen, Kees van Limpt, Jan Knol, Àngels Franch, Margarida Castell, Maria J. Rodríguez-lagunas, Francisco J. Pérez-Cano

**Affiliations:** 1 Departament de Bioquímica i Fisiologia, Facultat de Farmàcia i Ciències de l’Alimentació, University of Barcelona (UB), Av. Joan XXIII 27-31, 08028 Barcelona, Spain; mmrigo@gmail.com (M.d.M.R.-A.); angelsfranch@ub.edu (À.F.); margaridacastell@ub.edu (M.C.); franciscoperez@ub.edu (F.J.P.-C.); 2 Institut de Recerca en Nutrició i Seguretat Alimentària (INSA), C/ Prat de la Riba 171, Santa Coloma de Gramanet, 08921 Barcelona, Spain; 3 Danone Nutricia Research, 3584 Utrecht, The Netherlands; Karen.Knipping@danone.com (K.K.); j.garssen@uu.nl (J.G.); Kees.vanlimpt@danone.com (K.v.L.); jan.knol@danone.com (J.K.); 4 Division of Pharmacology, Utrecht Institute for Pharmaceutical Sciences, Faculty of Science, Utrecht University, 3512 Utrecht, The Netherlands

**Keywords:** fermented formula, probiotic, prebiotic, postbiotic, rotavirus

## Abstract

Several microbial modulatory concepts, such as certain probiotics and prebiotics, confer protection against gastrointestinal infections, among which is acute diarrhea caused by the rotavirus (RV). Other microbiota modulators, such as postbiotics, produced during fermentation, might also have the potential to counteract RV infection. In light of this, a fermented milk, made by using *Bifidobacterium breve* C50 (BbC50) and *Streptococcus thermophilus* 065 (St065) with a prebiotic mixture—short chain galactooligosaccharides/long chain fructooligosaccharides (scGOS/lcFOS 9:1)—with potential to impact the intestinal microbiota composition was tested. An RV infected rat model was used to evaluate the amelioration of the infectious process and the improvement of the immune response induced by the fermented milk with prebiotic mixture. The dietary intervention caused a reduction in the clinical symptoms of diarrhea, such as severity and incidence. Furthermore, a modulation of the immune response was observed, which might enhance the reduction of the associated diarrhea. In addition, the fermented milk with prebiotic mixture was able to bind the virus and reduce its clearance. In conclusion, the postbiotic components in the fermented milk in combination with the prebiotics used here showed protective properties against RV infection.

## 1. Introduction

The rotavirus (RV) is the leading worldwide etiological agent of severe dehydrating diarrhea in childhood under the age of five years [1]. RV infects and replicates mainly in the nondividing mature enterocytes near the tips of the small intestinal villi [2]. As a result, infants develop diarrhea, vomiting and fever for three days to one week, usually with reinfections, which tend to be less severe than the first infection [3]. The most common treatment consists of oral rehydration solutions (ORS). Prevention by vaccination also exists, although its efficacy is lower in some African countries [4]. Therefore, several nutritional interventions with bioactive compounds have been studied in recent years. Among these compounds, probiotics have been the most widely studied due to their known immunomodulatory effects and direct action, often on the small intestine, which is the primary point of the RV infection [5,6,7,8].

Probiotics are live microorganisms that, when administered in adequate amounts, confer a health benefit on the host [5]. These beneficial bacteria may directly interact with the host or the pathogen and even modulate the commensal microbiota, altogether preventing the infection of pathogens such as the RV. Several studies conducted over the last decade using probiotics, mainly from the *Lactobacillus* and *Bifidobacterium* genera, have showed effectivity against RV infection and symptoms through different mechanisms of action [6,7,8].

Prebiotics are defined as “substrates that are selectively utilized by host microorganisms conferring a health benefit” [9,10]. At the moment, there is only limited evidence based on a few *in vitro, in vivo*, and clinical interventions studying the effect of prebiotics as anti-RV agents, and mostly combined with probiotics as synbiotic mixtures. In view of this, several mechanisms have been proposed for the action of specific prebiotics against intestinal pathogens, including the RV, which involve both microbiota-dependent and -independent actions that can lead to direct and indirect effects on the virus (e.g., pathogen binding or immunostimulation of the host, respectively) [6,11]. One example of this is a specific prebiotic mixture based on short chain galactooligosaccharides (scGOS) and long chain fructooligosaccharides (lcFOS) in a 9:1 ratio, which has proven to be effective in previous preclinical studies of RV-associated diarrhea, alone or in combination with other components [6,11].

In the last few years, some studies have demonstrated that probiotic bacteria viability is not necessary to induce health-promoting effects, and that fractions and extracts from these bacteria and produced by these bacteria can be involved in such actions [8,12]. In this context, the term ‘postbiotic’ has been coined (defined as “bioactive compounds produced by food-grade microorganisms in a fermentation process”). Specific postbiotics are described as having biological activity in the host [8,13,14].

Our hypothesis is that in the context of early life, when a newborn is still immunologically immature, infant formula with a combination of specific postbiotics and prebiotics could be a strategy to prevent early life RV infections when breastfeeding is not possible. The present study aimed to examine the effects of a fermented milk formula (with postbiotics) and prebiotics on the protective effects against RV infection in an RV infected neonatal rat model.

## 2. Materials and Methods

### 2.1. Animals and Experimental Design

Nine G14 pregnant Lewis rats were obtained from Janvier (Le-Genest-Saint-Isle, France) and housed individually in cages (2184L Eurostandard Type II L, Tecniplast, West Chester, PA, USA) with large fibrous particle bedding and tissue paper as enrichment. They were monitored daily and allowed to deliver at term. The day of birth was registered as day 1 of life. Litters were unified to seven pups/dam with approximately a 1:1 ratio of males:females in each nest. The pups had free access to the nipples and the rat diet. Dams had access to water and the commercial diet (Teklad Global Diet 2014, Envigo, Indianapolis, IN, USA) corresponding to the American Institute of Nutrition (AIN)-93G diet (30) ad libitum. Each dam and its litter were housed individually under controlled temperature and humidity conditions, in a 12 h:12 h light:dark cycle. Furthermore, the animals were located in a special safe isolated room, specially designed and authorized for working under biosecurity level 2 conditions in the Faculty of Pharmacy and Food Science animal facility, University of Barcelona.

Once a day, after separating all the pups from their mother by placing the mother in a separate cage during the procedures, the pups were individually identified by labeling them with a permanent marker. The animals were weighed and monitored in order to obtain data regarding the influence of virus inoculation, clinical development, and the nutritional intervention on body weight and growth. Daily, after these interventional actions the whole litter was reunited together with the dam. Animal procedures were carried out at the same time period (during the light phase) each day to avoid variation in disturbance and effects on the day/night rhythm. This study was conducted in accordance with the institutional guidelines for the care and use of laboratory animals established by the Ethics Committee for Animal Experimentation of the University of Barcelona and the Catalonian Government (CEEA-UB ref. 165/11, DAAM: 5871) in full compliance with national legislation following the EU-directive 2010/63/EU for the protection of animals used for scientific purposes.

Suckling rats were randomly distributed in three different experimental groups: Reference (REF group), rotavirus (RV group), and the group receiving a heat-treated fermented milk and the prebiotic mixture scGOS/lcFOS in a 9:1 ratio, constituting the Fermented Milk + Prebiotic group (FM + P group). Each group was composed of three (mixed-sex) litters with seven pups each (*n* = 21/group). Diets or vehicle were oral administered from day 3 of life to the last day of the experiment. The RV inoculation was carried out at day 7 of life in both the RV and the FM + P group, except for the REF group. The REF group received the same volume of vehicle and was handled by the same experimental procedures.

SA-11 infection was evaluated on days 8–21 by the growth rate and clinical indexes derived from fecal samples as previously described [15]. Fecal sampling was performed once a day by gently pressing and massaging the abdomen. Specimens were immediately scored, in a blinded manner by at least three different researchers (as described in Section 2.4 Clinical Indexes), weighed, and frozen at −20 °C for further analysis.

Animals were randomly euthanized at two different time points: Three animals of each litter were euthanized 1 week after the RV inoculation, on day 14 (*n* = 9/group), while the other four animals of each litter continued receiving the diet until the end of the suckling period, on day 21 (*n* = 12/group). At the day of sacrifice, animals were intramuscularly anesthetized with ketamine (90 mg/kg) (Merial Laboratories S.A., Barcelona, Spain) and xylazine (10 mg/kg) (Bayer A.G., Leverkusen, Germany) and blood was collected by cardiac puncture for hematocrit (HCT) determination and sera obtention, which was stored at −20 °C until further analysis. After the death of the animals, liver, spleen, large intestine, and small intestine were dissected and weighed. In addition, the small intestine was cut into 5 mm pieces and incubated with PBS for 10 min at 37 °C in a shaker to obtain the gut wash (GW) [15]. After centrifugation, supernatants were stored at −20 °C until analysis.

### 2.2. Dietary Supplementation

Animals were administered daily from day 3 to day 21 of life with the fermented milk/prebiotic mixture (FM + P group) or vehicle (RV and REF groups) by oral gavage, as previously described [16], using low-capacity syringes (Hamilton Bonaduz, Bonaduz, Switzerland) adapted to forced alimentation tubes of 25 or 23 caliber and 27 mm in length (ASICO, Westmont, IL, USA).

The fermented milk was obtained by fermentation of a milk matrix with *Bifidobacterium breve* C50 and *Streptococcus thermophilus* 065 during the manufacturing process (Blédina, France). This process added to the composition of the formula active bacterial metabolites and cell fragments (postbiotic compounds). The prebiotic supplement consisted of a combination of scGOS/lcFOS mixture in a 9:1 ratio (Nutricia, the Netherlands). The animals from the FM + P group received a dose of a mixture of both concepts at a dose of 3 g of the postbiotic and 0·8 g of the prebiotic/100 g of body weight/day.

### 2.3. Virus Inoculation

The RV strain selected for the infection (simian SA-11) was produced by the ‘Enteric Virus Group’ of the University of Barcelona, as in previous studies [6,15]. Briefly, propagation of viruses was done in fetal African green monkey kidney cells (MA-104) and tittered as TCID_50_/mL (TCID, tissue culture infectious dose). Viruses were produced in compliance with the current principles of Good Laboratory Practices (Royal Decree 1369/2000, of 19 July). SA-11 was orally inoculated (2 × 10^8^ TCID_50_/rat in 100 μL of phosphate-buffered solution, PBS) at day 7 of life in suckling rats from the RV and FM + P groups. The RV inoculations were performed 1 h after separation from their dams to avoid interference between RV and milk components, following previous procedures from the research group [15]. The REF group received the same volume of PBS applying the same procedures under similar conditions.

### 2.4. Clinical Indexes

The severity of diarrhea was evaluated by both the fecal weight and by scoring stools. Fecal specimens were scored in a blinded manner from 1 to 4 (diarrhea index, DI) based on color, texture, and amount according to the following scale: Normal (diarrhea index = 1), loose yellow-green (diarrhea index = 2), totally loose yellow-green (diarrhea index = 3), high amount of watery (diarrhea index = 4) feces. A diarrhea score ≥2 indicates diarrheic feces whereas scores of diarrhea index <2 indicate absence of diarrhea. In addition, to find a global value of severity of diarrhea the analysis of the area under the curve (AUC) of the severity during the diarrhea period (days 7–13) was performed. The maximum diarrhea index was defined as the highest score during the diarrhea period.

Incidence of diarrhea was expressed by the % of diarrheic animals (%DA), considering the number of animals in each group, and by the % of diarrheic feces (%DF), considering the number of total samples collected every day in each group. The AUCs of the % of diarrheic animals and the % of diarrheic feces during days 7–13 were calculated as a global value of incidence. The maximum % of diarrheic animals and diarrheic feces were defined as the highest values during the diarrhea period. The diarrhea period was calculated for each animal as the interval between the first and the last day of diarrhea. The actual days with diarrhea within the diarrhea period were also counted.

The mean values and AUCs for severity, %DA and %DF were also calculated considering the basal values due to intrinsic aspects of each treatment (normalized results) as in previous studies [17].

Frozen fecal samples from days 8−12 were diluted up to 200 mg/mL in distilled water, softly agitated and then the pH was measured by a pH electrode for surfaces 5207 couple to a pH-Meter micropH 2001 (Crison Instruments, Barcelona, Spain).

### 2.5. ELISA for Specific Anti-RV Humoral Response

Following previous procedures [16], UV-inactivated SA-11 particles at 10^5^/mL were used for coating the 96-well plates (Nunc MaxiSorp, Wiesbaden, Germany). After blocking with PBS-1% bovine serum albumin (BSA, 1 h, room temperature (RT)), appropriate diluted sera or intestinal wash samples were added (3 h, RT). Rabbit anti-rat Ig conjugated to peroxidase from Dako (Barcelona, Spain) or mouse biotinylated anti-rat IgA (A93-2), IgG1/2a (R19-15) or IgM (G53-238) monoclonal antibodies (MAb) from BD Biosciences (Heidelberg, Germany) were added after washing. Afterwards, peroxidase-conjugated extravidin (Sigma–Aldrich, Madrid, Spain) and substrate were added. Pooled sera from RV-infected animals from previous studies were used as control positive in each plate. Quadratic polynomial adjustment was used. The concentration of total anti-RV Ab and anti-RV IgG, IgA and IgM Abs from the RV group received the value of 100 arbitrary units (AU)/mL and the results of the FM + P group and those from day 21 were expressed with respect to this given value.

### 2.6. Quantification of SA-11 Viral Load in Feces and RV Blocking Activity

Fecal samples from up to 10 days after virus inoculation (7–16 days of age) were diluted in PBS at 20 mg/mL and homogenized using a Polytron (Kinematica, Luzern, Switzerland). Fecal homogenates were centrifuged (200 *g*, 5 min, 4 °C), and supernatants were frozen at −20 °C until use. Viral particles in fecal homogenates were quantified by a previously described ELISA technique [11,15]. SA-11 virus particles (10^5^ to 10^3^/mL) were used as the standard in each plate.

An in vitro blocking assay used in previous studies was performed to test the ability of the mixture to bind to the SA11 virus [11]. Briefly, a 96-well plate was co-incubated with 5 × 10^4^ particles/mL of SA-11 in PBS-Tween 1% with different dilutions of the in vivo-administered concentration of the product (from 1/2 to 1/60 dilutions) and incubated for 30 min. The unbound viral particles were quantified by ELISA, as described above (SA-11 viral load).

### 2.7. Statistical Analysis

The Appraising Project Office’s program from the Universidad Miguel Hernández de Elche (Alicante) allowed the calculation of the sample size considering the number of pups as the statistical unit and the clinical outcome as the main parameter. The number of animals in each group was established in order to allow the detection of statistically significant differences among groups assuming that there was no dropout rate and a type I error of 0.05 (two-sided).

For the statistical analysis, the PASW Statistics 20 software package (SPSS Inc, Chicago, IL, USA) was used. Komolgorov−Smirnov and Levene’s tests were applied to assess normal distribution and variance equality, respectively. Conventional one-way ANOVA was performed considering the experimental group as the independent variable. When SA-11 inoculation/treatment had a significant effect on the dependent variable, Scheffé’s post hoc test was applied. Kruskal−Wallis and Mann–Whitney U tests were used when non-normal distribution or different variance were found. Finally, the chi-square test was used to compare diarrhea incidence. Differences were considered significant at *p* values of <0·05. All the results are expressed as mean and SEM of number of animals.

## 3. Results

### 3.1. Effect of the Dietary Supplementation and Virus Inoculation on Morphometric Variables

**Figure 1:**
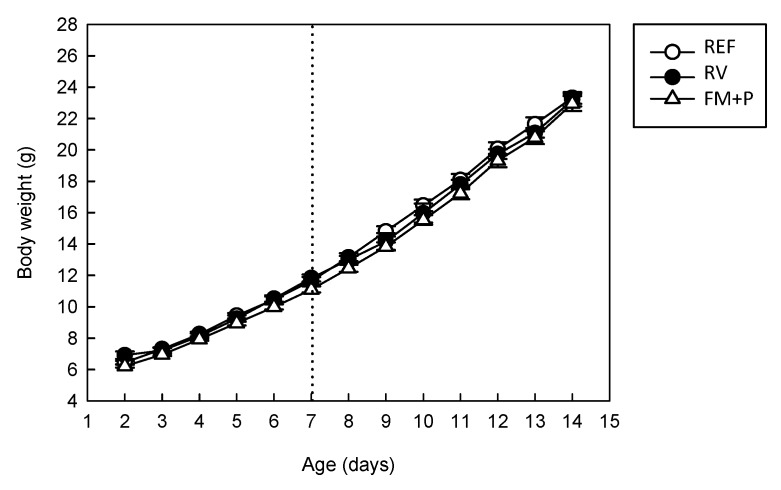
Body weight (g) from the Reference (REF), the Rotavirus (RV) and the Fermented Milk + Prebiotic (FM + P) groups throughout the first 14 days of the study. The period includes before and after virus inoculation on day 7 (dashed vertical line). Results are expressed as mean values ± SEM (*n* = 21 animals/group). No statistical differences were found.

Body weight was recorded throughout the 14 days of intervention in all animals and neither the diet nor the viral infection influenced their growth (Figure 1). The initial weight, measured on the second day of life, was around 6 g (6.92 ± 0.24 g for REF, 6.45 ± 0.10 for RV, 6.22 ± 0.10 for FM + P) without differences among groups. Body weight on inoculation day (day 7 of life) was still similar among groups (11.66 ± 0.24 g for REF, 11.83 ± 0.22 for RV, 11.10 ± 0.20 for FM + P), showing no diet effect. Moreover, the absence of differences after the diarrhea period, at one week post-inoculation (day 14), meant that body weight loss due to infection or diet influence can be disregarded (23.32 ± 0.37 g, 23.17 ± 0.41 g, 22.96 ± 0.48 g for REF, RV and FM + P groups, respectively).

However, at the end point of the experiment at day 21 (*n* = 9/group), some differences appeared in the RV group: A significant decrease in body weight (28.76 ± 0.42) was observed compared to the REF group (34.6 ± 0.66 g, *p* < 0.05), which was prevented by the FM + P diet (36.11 ± 0.40 g). If the overall body weight gain (% from day 2 to 21) is considered, this body weight loss induction in the RV group (*p* < 0.05) and the protection by the FM + P intervention is still observed: 411.11 ± 11.13 g for REF, 310.45 ± 19.63 g for RV and 439.90 ± 4.44 g for FM + P group.

The rotavirus infection also induced some changes on organ weight on both day 14 and 21 (Table 1). Specifically, 7 days after infection (day 14 of life) the RV infection induced a lower spleen and higher liver relative weight (*p* < 0.05 vs. REF).

The effect on the weight of the liver was also observed in the FM + P group, however the FM + P administration induced an increase in the relative weight of the large intestine (LI) and the small intestine (SI) (*p* < 0.05 vs. REF and RV groups). The results from day 21 evidenced a significant impact on the RV group with lower relative weight of spleen, liver and SI compared to the REF group (*p* < 0.05). This was not the case for the FM + P group, where the supplementation with the FM + P mixture prevented the impact of the RV infection on those variables (*p* < 0.05 vs. RV).

### 3.2. Effect of the Dietary Supplementation on Stool Consistency

**Figure 2:**
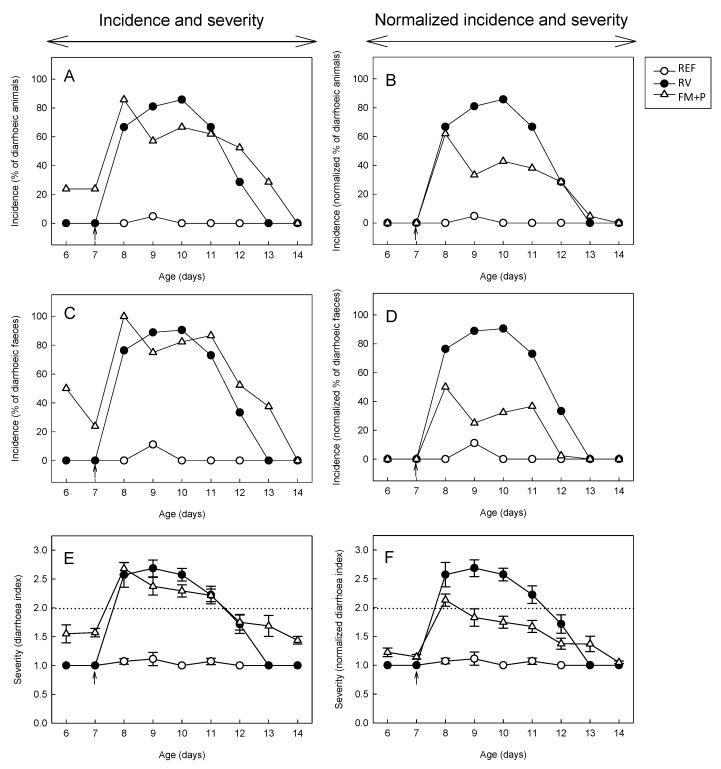
Diarrhea incidence and severity from the Reference (REF), the Rotavirus (RV) and the Fermented Milk + Prebiotic (FM + P) groups. Percentage of diarrheic animals (% DA) before (**A**) and after subtracting the basal values due to intrinsic aspects of the treatment (**B**); percentage of diarrheic feces (% DF) before (**C**) and after subtracting the basal values due to intrinsic aspects of the treatment (**D**); severity of diarrhea in a scale from 0 to 4 before (**E**) and after (**F**) subtracting the basal values due to intrinsic aspects of the treatment. DI ≥ 2 indicates diarrheic feces. Values are means (*n* = 21 animals/group) ± SEM. Statistical significance is explained in the text.

The severity and incidence of diarrhea during the studied period (from day 7 to day 14) are shown in Figure 2. The FM + P administration itself had a direct impact on fecal consistency, increasing the DI for the fecal specimens and the number of feces considered as diarrheic (DI ≥ 2) (Figure 2A,C,E). This effect, already described for certain prebiotics and in previous studies in our laboratory, such as the scGOS/lcFOS [11], was taken into account to normalize the results (Figure 2B,D,F).

### 3.3. Incidence of Diarrhea

Two different approaches to evaluate the incidence of diarrhea were used: % of diarrheic animals (% DA) and % of diarrheic feces (% DF). The % DA showed that almost all the RV-inoculated animals, 95–100%, displayed diarrhea at sometimes. As expected, the animals from the REF group (with no RV inoculation) showed < 5%diarrhea.

The % DA in the RV group was higher than 80% during the first 4 days immediately after the SA-11 inoculation (from day 8 to day 11 of life). After that, this percentage decreased, and on day 13 none of the animals in the RV group had diarrhea-like feces (Figure 2A). The animals from the FM + P group displayed some improvement during the diarrhea period. Specifically, this group showed a trend towards a lowering of the % DA with respect to the RV group on the following two days after the induction of the infection (Figure 2A). However, as expected, the diarrhea incidence of the FM + P group tended to be even higher than that of the RV group before virus inoculation, the day after infection (day 8) and in the last period studied (days 12−13). These results indicated an intrinsic fecal loosening effect of the FM + P, and for that a normalization was used, as explained above. This resulted in a clearer amelioration of the diarrhea incidence, which was significantly lower than that of the RV group on days 9 and 10 of life (*p* < 0.05, Figure 2B).

The calculation of diarrhea incidence as the % DF, which only takes into account the number of diarrheic feces with respect to the fecal specimens obtained, displayed the same pattern as that of the above results for all groups (Figure 2C). In fact, the intrinsic impact on fecal consistency of the FM + P product was more evident here, as can be observed in the proportion of 55 to 25% DF for the two days previous to the SA-11 inoculation, showing that the normalization of the results elucidated an even more effective impact on the amelioration of diarrhea (Figure 2D).

The diarrhea process in the RV group started 1–2 days after inoculation and ended around day 11 of life. Both the diarrhea period and the days with diarrhea occurred between 3 and 4 days. In the FM + P group, the length of these periods were significantly increased when no normalization was applied (*p* < 0.05) and it can be observed that the FM + P group still had scores >1 up to the end of the study in the case of the % DA but not for the % DF (Figure 2A,C, respectively). Normalization again leads to the observation of a shortening of the diarrhea period, especially in the % DF (Figure 2D).

**Figure 3:**
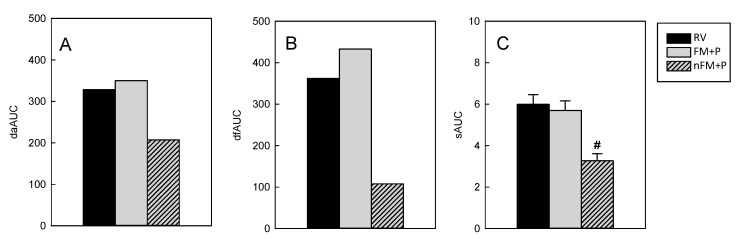
Overall diarrhea incidence and severity AUCs. AUC of the incidence expressed as percentage of diarrheic animals (daAUC) (**A**), or of diarrheic feces (dfAUC) (**B**), and AUC of the severity pattern (sAUC) (**C**). Results are shown for animals from the Rotavirus (RV) group (black bars), Fermented Milk + Prebiotic (FM + P) group (grey bars) and Fermented Milk + Prebiotic group after subtracting the basal values due to intrinsic aspects of the treatment (nFM + P) (grey striped bars). As Reference animals did not develop diarrhea, they had AUC values equal to 0 (data not shown). Results are expressed as the overall incidence pattern corresponding to all animals together or the mean of each individual severity pattern from the 21 animals/group. Standard errors are shown in Figure 3C. Statistical significance: # *p* < 0.05 vs. RV.

In any case, the RV group had the maximum incidence (% DA and % DF) 3 days after the RV induction (day 10), whereas the FM + P group displayed the highest value the first day post-infection (day 8), a fact that is better observed in Figure 2B,C. When both the % DA-AUC and the % DF-AUC were calculated (Figure 3A,B, respectively), the RV group had values of 300−400 arbitrary units whereas REF animals did not develop diarrhea and had AUC values around 0. It can also be observed that the FM + P group did not present a lower value than that of the RV group, however, when the AUC from both types of incidence was calculated from normalized values in the FM + P group (nFM + P) a clear overall reduction was observed (Figure 3A,B).

### 3.4. Severity of Diarrhea

The severity curves before (Figure 2E) and after (Figure 2F) normalization show an amelioration of the process by the FM + P intervention. The DI in the RV group was higher than 2.5 from day 8 to day 10, after which the mean score descended to approximately 2 on day 11 and even lower thereafter, indicating absence of diarrhea in most of the animals. The FM + P group showed a tendency to lower the severity score more than the RV group during the first 2 days after the inoculation (day 8) (Figure 2E) however, a significative reduction was only observed after normalization (Figure 2F), when DI < 2 are observed in all post-infection days with exception of the first day.

The mean maximum DI for both infected groups was around 3 and was obtained at day 9 in all cases. Specifically, the FM + P group had a mean maximum score of 2.82 ± 0.09 on a mean day of 8.57 ± 0.34, whereas the RV group showed a trend towards having a higher maximum index (3.05 ± 0.11), which appeared later in time (on a mean day of 9.00 ± 0.25).

The AUC of the severity pattern (sAUC) was about 6 in both the RV and the FM + P groups (Figure 3C), without significant differences between them, but again, a significant reduction in sAUC (~50%) was observed for the nFM + P group compared to the RV group, demonstrating an overall reduction in the severity of the process when intrinsic features of the product are taken into account (*p* < 0.05).

### 3.5. Fecal Weight

**Figure 4:**
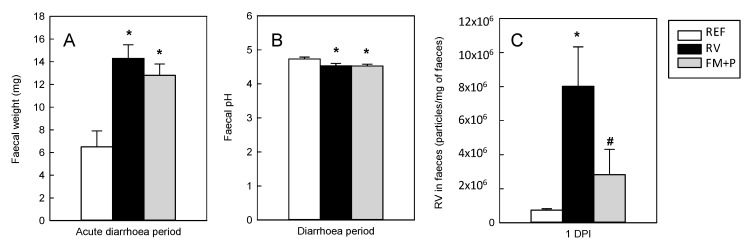
Diarrhea and infection indicators from the Reference (REF), the Rotavirus (RV) and the Fermented Milk + Prebiotic (FM + P) groups. Mean fecal weight through the early post-infection period (days 8−11) (**A**); mean fecal pH during same diarrhea period (**B**); and viral shedding on day 1 post-infection (DPI) (**C**). Results are shown for animals from the REF group (white bars), RV group (black bars) and FM + P group (grey bars). Results are expressed as mean and SEM of samples or determinations derived from 21 animals/group. Statistical significance: * *p* < 0.05 vs. REF, # *p* < 0.05 vs. RV.

The fecal weight of samples throughout the early post-infection period (days 8−11) is an objective measure of water incorporation in the feces due to the diarrhea. A significant positive correlation exists between the fecal weight (objective measure) and the assigned DI (subjective measure) for each specimen (Pearson coefficient of 0.633, *p* < 0.05). Mean fecal weight from samples obtained before the inoculation day (day 7) was similar among the groups (from 3.2 to 4.6 mg/specimen). The day immediately after the inoculation (day 8), the fecal weight of animals from the RV group (20.2 ± 4.0 mg/sample) increased significantly compared to that of the REF group (4.3 ± 0.8 mg/sample, *p* < 0.05) and this diarrhea indicator was counteracted by the FM + P administration (13.7 ± 2.4 mg/sample from the FM + P group). However, when all samples from the acute diarrhea period were considered together, the three times higher fecal weight from the RV group with respect to those from the REF group (*p* < 0.05) was not modulated in the FM + P group (Figure 4A).

### 3.6. Fecal pH

The pH of fecal samples was measured from each group in the early post-infection period (days 8−11), when diarrhea was still present in all infected groups (Figure 4B). The RV group displayed a lower fecal pH in all days during the diarrhea period in comparison with the REF group (*p* < 0.05), however no correlation was found between pH and fecal weight or DI (Pearson coefficient <0.1 in both cases). In addition, the FM + P intervention was not able to significantly avoid the acidification of feces due to the diarrhea induced by the SA-11 inoculation. Only the lower pH induced by the diarrhea on day 8 (4.64 ± 0.07 and. 4.72 ± 0.10 for RV and REF groups) and day 13 (4.25 ± 0.09 and. 4.73 ± 0.15 for RV and REF groups, respectively, *p* < 0.05) was controlled by the diet both on day 8 (4.74 ± 0.06) and on day 13 (4.79 ± 0.25, *p* < 0.05 vs. RV).

### 3.7. Viral Shedding and In Vitro Blocking Assay

The viral shedding was evaluated after inoculation (days 7−16). In all RV-inoculated animals, the maximum viral elimination (*p* < 0.054 vs. REF) occurred on the first day after inoculation (day 8, Figure 4C), whereas no differences were observed between the REF and RV groups afterwards. On the first day after inoculation, the FM + P intervention partially avoided such shedding increase, showing a lower viral load than the RV group (*p* < 0.05) and similar to the REF animals.

The reduction in the viral shedding by the FM + P supplementation led to the analysis of the tested product’s capacity to bind to RV particles using an in vitro approach. This assay allows the quantification of the number of SA-11 particles detected after incubation with several dilutions of the product as well as the percentage of inhibition. All product dilutions from 1/2 to 1/30 from the one used in the intervention have an inhibitory activity of around 90%, indicating a high blocking potential of this mixture.

### 3.8. Humoral Immune Response

**Figure 5:**
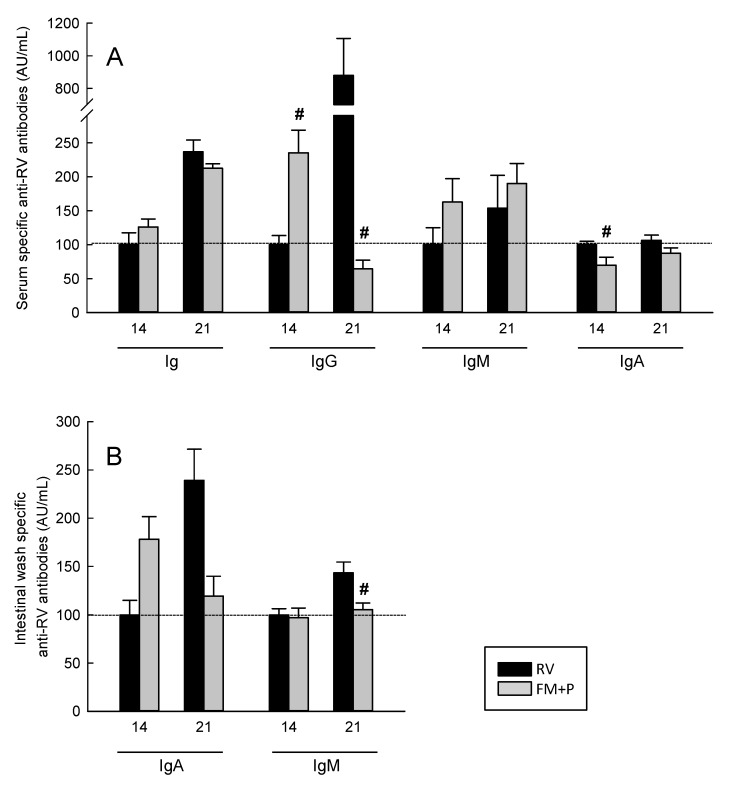
Specific anti-RV antibodies in serum (**A**) and intestinal wash (**B**) from 14- and 21-day-old rats. The values of each isotype in the Rotavirus (RV) group on day 14 are considered as 100 AU, and the results for the Rotavirus (RV) group and for the Fermented Milk + Prebiotic (FM + P) group on day 21 have been proportionally adjusted for each isotype. Results are expressed as mean and SEM (*n* = 9−12 animals/group) in AU/mL. # *p* < 0.05 vs. RV group.

Serum-specific anti-RV antibodies (total, IgG, IgM and IgA) (Figure 5A) and intestinal IgA and IgM (Figure 5B) were evaluated at day 14 and 21 of life. The RV group had measurable levels of the specific anti-RV total, IgA, IgG and IgM in serum and gut wash even at day 14 of life.

Dietary supplementation with FM + P was able to modify the specific humoral immune response against the RV. On day 14, whereas the total serum anti-RV antibodies seemed not to be affected by the diet, a trend towards an increase of IgM, a significant increase of IgG (*p* < 0.05 vs. RV) and a significant decrease of IgA (*p* < 0.05 vs. RV) was observed. On day 21, only the anti-RV IgG was modified by the diet, showing a clear reduction in the FM + P group (*p* < 0.05) (Figure 5A).

Regarding the intestinal compartment, although there was a trend to induce higher levels of IgA on day 14 but lower levels on day 21 due to the FM + P diet, the only significant impact was on anti-RV IgM levels on day 21, which were lower than those from the RV group (*p* < 0.05).

## 4. Discussion

In the present study the combination of a specific fermented milk (providing specific postbiotic compounds) and the prebiotic mixture (scGOS/lcFOS) showed the capacity to ameliorate the RV-diarrhea in a model of suckling rats. The prebiotic enriched fermented formula reduced the incidence and severity of the diarrhea process. In addition, the combination was able to directly interact with the virus and to enhance the host immune system.

The rationale for selecting both microbial modulator concepts was based on the following studies. On the one hand, the specific prebiotic mixture based on short-chain galactooligosaccharides (scGOS) and long-chain fructooligosaccharides (lcFOS) in a 9:1 ratio resembles human milk oligosaccharide structures and is frequently added to infant formulas. This formula has been described to induce changes in microbiota composition and fecal consistency comparable to those of infants receiving human milk [18]. It increases the production of short chain fatty acids (SCFAs) in caecum samples and intestinal IgA levels [19,20], altogether leading to a lower incidence of infections and allergic diseases in infancy in human intervention studies [21,22,23]. It is also effective against RV infection at a preclinical level [6,11]. On the other hand, foods fermented by specific bacterial strains, and as a result containing bioactive compounds that have been produced during the fermentation process can lead to beneficial properties [12]. The strains used for the fermentation were *Bifidobacterium breve* C50 (*Bb*C50) and *Streptococcus thermophilus* 065 (*St*065) because their metabolites have been shown to modulate intestinal microbiota composition in animal and clinical studies by promoting bifidobacteria population [24,25]. In addition, this fermented formula has evidenced a potential to modulate acute diarrhea in preclinical and clinical studies [17,26].

The nutritional intervention with the combination of prebiotics and postbiotics showed relevant biological changes in the growth of the small and large intestine after 14 days and on body weight after 21 days of supplementation compared to the REF and RV groups. In addition, an intestinal trophic effect was also observed by other prebiotics, which showed not only an increase in the intestine weight but also an enhancement of the intestinal structure [27,28]. Furthermore, this effect is also consistent with another study showing the impact of postbiotics derived from lactobacilli on the intestinal architecture of broilers [29]. Overall, the present study showed intestinal growth-promoting effects that could be due to improved intestinal maturation following this fermented milk−prebiotics mixture supplementation. Finally, in relation to the effect on other organs, we have found some effect on liver and spleen weights due to the mixture used that could be linked to the influx/efflux of immune cells to infection. This result agrees with another study that used the same fermented formula and found an increasing effect on thymus size from healthy term infants in comparison with babies fed with a standard infant formula [30].

Regarding the diarrhea, the softer consistency of fecal samples induced by scGOS:lcFOS has been described in previous studies [11]. This property, already seen in several other prebiotics, brings the fecal consistency of formula-fed babies to that of breast-fed [18,31]. However, in this model this fact masks the associated diarrhea process. Therefore, during the post-inoculation period, although some reduction in both diarrhea incidence and severity, as well as fecal weight, was seen, only a statistical effect was found after considering these basal properties.

Besides the diarrhea as a clinical symptom, the RV inoculation also induced an electrolytic disorder responsible for the intestinal pH reduction shown herein and in previous studies [6,11]. This alteration seems to be due to the disruption of the tight junction and the secretion of chloride to the lumen, as well as the activation of the enteric nervous system due to RV virions and proteins, such as NSP4 [7]. The intervention with the combination of prebiotics and postbiotics was not able to prevent a decrease in the pH, although the components of the mixture alone did show this effect in previous studies [6,11]. Furthermore, the postbiotic’s ability to inhibit chloride secretion previously demonstrated in vitro [32] was not observed in this mixture. However, the fact that the postbiotic did not prevent fecal acidification could be explained by its direct effect on reducing the stools’ pH, as described by the study of Indrio and collaborators [30].

The lower viral shedding found in this study following the intervention enable us to suggest that the components assayed may have a binding effect with the virus. On the one hand, the supplementation partially avoided on the one hand the viral infection, and on the other hand the detection of the virus using the ELISA technique. This finding is in line with previous results with similar components [11,17], where the same in-house blocking assay was used to confirm this hypothesis. This microbiota-independent anti-infective effect of prebiotics or postbiotics involves direct interactions with pathogens and is associated with receptor mimicry and is already known for some types of dietary carbohydrates [33]. Thus, in our model, some components of the mixture administrated may act as soluble receptor analogues for specific molecules of the host to which the virus could adhere. Therefore, this interaction between the pathogen and the products dislodges the adherent pathogen from the gut, reducing the infectivity of the RV, thus reducing the diarrhea severity.

Very few studies have addressed the immunomodulatory or intestinal protective role of fermented infant formula. Metabolic products derived from *lactobacilli* have been studied with special attention. To date, postbiotics derived from *L. rhamnosus* GG are able to protect human colonic smooth muscle cells from lipopolysaccharide (LPS) damage, modulate intestinal permeability and prevent cytokine-induced apoptosis [12,34,35,36]. Heat-labile metabolites of *L. paracasei* have also shown some beneficial actions on tissue integrity [37], peptidoglycan derived from a specific *L salivarius* strain protected mice from TNBS-induced colitis [38] and postbiotics derived from *L casei* DG reduce ex vivo mucosal inflammation [39]. However, the activity of bioactive compounds from *Bifidobacterium* strains and other bacteria has also been studied [12]. Interestingly, each bacterial fraction (whole cell, cell-free extracts, purified cell walls and culture supernatants) of *Bifidobacterium bifidum BGN* displayed different immunological actions [40]. Thus, the metabolites produced through the natural process of fermentation may be responsible for the results in this study. The variety of these compounds may vary, from lactic acid, bacteriocins or other antibacterial substances, oligosaccharides and glycoproteic complexes produced during lactic fermentation as well as DNA and bacterial wall fractions released in the fermentation or inactivation processes.

Independently of the compound/s involved, we cannot discard that one of the mechanisms participating in the modulatory effects of this combination of compounds could be the stimulation of the endogenous microbiota, which may involve the bifidogenic effect and eventual enhancement of immune responses against the virus [14]. In fact, both the scGOS:lcFOS mixture [6,11] and the postbiotic used here have demonstrated their immunomodulatory activity in previous studies [17,41]. Although the changes in anti-RV Ab are somewhat difficult to interpret, it seems that the mixture enhances the early response (day 14) in terms of sera anti-RV IgG and intestinal anti-RV IgA, which have an impact in the late immune response (day 21). This hypothesis is in line with the modulatory action of scGOS:lcFOS in a double model of infection [6]. In this line, this fermented formula in partially breast-fed infants induced higher fecal IgA compared with those fed the standard formula [42] and in immunized mice lead to higher specific Ab titers than control fed mice [41].

Moreover, a clinical study using this postbiotic formula suggested that its immunomodulatory action may be due to the presence of thermoresistant peptidoglycans contained in the cell wall of the ferments that could activate the toll-like receptor (TLR)-2 [43]. In addition, another mechanism of action for some postbiotic compounds refers to the induction of the intestinal epithelial homeostasis by involving several cellular signaling pathways [12,14], and for this particular fermented formula, strengthening the intestinal barrier [41]. Thus, although we have not studied this particular mechanism, it could also be involved in the protective role against the RV shown here for the fermented milk−prebiotics combination.

Future research should be directed to the specific mechanisms responsible for this beneficial effect, particularly the blocking of the pathogen and the immunomodulatory action.

## 5. Conclusions

In conclusion, the results obtained in the present study emphasize the potential of the combination of specific postbiotics and prebiotics in the prevention of RV-associated diarrhea at a preclinical level. Further studies are necessary to ascertain the precise mechanisms of these bioactive compounds. The use of the specific fermented milk with prebiotic mixture like the one used in this study could be an effective and safe strategy to include in infant formulas to help in the prevention of acute diarrhea in children.

## Figures and Tables

**Table 1:** Organ weight and hematocrit (HCT) from animals on day 14 and 21 of life.

		REF	RV	FM + P
**day 14**	**Spleen (%)**	0.50 ± 0.01	0.46 ± 0.02 *	0.50 ± 0.02
	**Liver (%)**	2.91 ± 0.03	3.48 ± 0.16 *	3.27 ± 0.15 *
	**Small intestine (%)**	3.53 ± 0.23	3.62 ± 0.14	4.86 ± 0.18 *#
	**Large intestine (%)**	0.34 ± 0.01	0.34 ± 0.01	0.39 ± 0.02 *#
	**HCT**	25.27 ± 1.53	22.54 ± 1.11	23.99 ± 1.62
**day 21**	**Spleen (%)**	0.38 ± 0.01	0.33 ± 0.01 *	0.37 ± 0.01 #
	**Liver (%)**	3.63 ± 0.24	2.62 ± 0.07 *	4.01 ± 0.11 #
	**Small intestine (%)**	4.03 ± 0.27	3.36 ± 0.10 *	3.97 ± 0.14 #
	**Large intestine (%)**	0.43 ± 0.02	0.39 ± 0.03	0.47 ± 0.04
	**HCT**	20.62 ± 2.50	19.37 ± 3.41	20.52 ± 2.11

Results are expressed as mean values ± SEM (*n* = 9−21 animals/group). REF, Reference group; RV, Rotavirus group; FM + P, Fermented Milk + Prebiotic group. Statistical significance: * *p* < 0.05 vs. REF, # *p* < 0.05 vs. RV.
